# Motor Unit-Driven Identification of Pathological Tremor in Electroencephalograms

**DOI:** 10.3389/fneur.2018.00879

**Published:** 2018-10-29

**Authors:** Aleš Holobar, Juan A. Gallego, Jernej Kranjec, Eduardo Rocon, Juan P. Romero, Julián Benito-León, José L. Pons, Vojko Glaser

**Affiliations:** ^1^Faculty of Electrical Engineering and Computer Science, University of Maribor, Maribor, Slovenia; ^2^Neural and Cognitive Engineering Group, Centre for Automation and Robotics, Spanish National Research Council, Arganda del Rey, Spain; ^3^Neurorehabilitation and Brain Damage Research Group, Experimental Sciences School, Universidad Francisco de Vitoria, Madrid, Spain; ^4^Brain Damage Unit, Hospital Beata María Ana, Madrid, Spain; ^5^Department of Neurology, University Hospital 12 de Octubre, Madrid, Spain; ^6^Center of Biomedical Network Research on Neurodegenerative Diseases, Madrid, Spain; ^7^Department of Medicine, Faculty of Medicine, Complutense University of Madrid, Madrid, Spain; ^8^Neural Rehabilitation Group, Cajal Institute, Spanish National Research Council, Madrid, Spain

**Keywords:** pathological tremor, EEG decomposition, surface EMG decomposition, Parkinsonian tremor, essential tremor

## Abstract

**Background:** Traditional studies on the neural mechanisms of tremor use coherence analysis to investigate the relationship between cortical and muscle activity, measured by electroencephalograms (EEG) and electromyograms (EMG). This methodology is limited by the need of relatively long signal recordings, and it is sensitive to EEG artifacts. Here, we analytically derive and experimentally validate a new method for automatic extraction of the tremor-related EEG component in pathological tremor patients that aims to overcome these limitations.

**Methods:** We exploit the coupling between the tremor-related cortical activity and motor unit population firings to build a linear minimum mean square error estimator of the tremor component in EEG. We estimated the motor unit population activity by decomposing surface EMG signals into constituent motor unit spike trains, which we summed up into a cumulative spike train (CST). We used this CST to initialize our tremor-related EEG component estimate, which we optimized using a novel approach proposed here.

**Results:** Tests on simulated signals demonstrate that our new method is robust to both noise and motor unit firing variability, and that it performs well across a wide range of spectral characteristics of the tremor. Results on 9 essential (ET) and 9 Parkinson's disease (PD) patients show a ~2-fold increase in amplitude of the coherence between the estimated EEG component and the CST, compared to the classical EEG-EMG coherence analysis.

**Conclusions:** We have developed a novel method that allows for more precise and robust estimation of the tremor-related EEG component. This method does not require artifact removal, provides reliable results in relatively short datasets, and tracks changes in the tremor-related cortical activity over time.

## Introduction

The role of cerebral cortex in the generation of pathological tremor has been widely studied in essential as well as in Parkinsonian tremor. Accumulated evidence suggests that tremor-related cortical activity exists in both types of tremor (1–5). Moreover, because of the significant coupling between the cortical activity and the activity in the affected muscles, motor cortex is thought to contribute to tremor generation (2, 3, 6–15).

To the best of our knowledge, all of the existing studies assessed corticomuscular coupling by computing the coherence between cortical activity, recorded with EEG or magnetoencephalograms (MEG), and an estimate of muscle activity derived from the surface EMG (2, 3, 6–13, 15, 16). Since the coherence function reveals a linear relationship between two signals at a given frequency (17), coherence at the tremor frequency is assumed to indicate tremor-related EEG activity. Although robust to noise at different frequencies, coherence only provides an indirect measure of corticomuscular coupling, and does not enable tracking changes in tremor properties over a short time scale. Furthermore, it requires off-line processing of relatively long EEG and EMG recordings, which need to be cleaned of artifacts beforehand. This limits the comparison of the tremor-related cortical activity across conditions and diseases.

Besides the coherence function, the cortical tremor component could also be potentially identified using blind source separation (BSS) algorithms. For example, Delorme et al. (18) identified, using independent component analysis (ICA) techniques, a number of components in the EEG activity of healthy subjects performing a working memory task. However, no group has demonstrated the feasibility of separating the tremor component from other brain activity. Notably, all the ICA/BSS algorithms proposed so far build on general assumptions about the EEG properties such as independence of the identified components, and would not exploit the specific characteristics of tremor, such as the relationship between sensorimotor cortical activity and muscle activity. As a result, they would suffer from large inter-trial and inter-subject variability in convergence toward the specific (tremor-related) EEG component.

With the exception of Gallego et al. (15), where we used cumulative motor unit spike trains (CST) to characterize the neural drive to the muscle, the authors of all of the studies mentioned above used the rectified surface EMG as an estimator of muscle activity. However, recent studies have shown that the CST of several (e.g., ≥5) motoneurons that innervate a muscle provide a more accurate representation of the synaptic inputs to a motoneuron pool than the EMG envelope (19–21). In addition, rectification of the surface EMG may or may not enhance the detection of synaptic inputs to the pool depending on the muscle contraction level (22). Indeed, as shown in Farina et al. (22) rectification is preferable over the raw EMG only at low contraction levels. Therefore, methods based on the CST rather than on the traditional surface EMG analysis are likely to identify tremor-related cortical activity more reliably.

In this study, we present and validate a novel method to identify tremor-related cortical activity. This method builds on the assumption that the tremor-related EEG component is stochastically phase-locked to the motor unit firings in a tremulous muscle. This implies close to linear relationship between the cortical activity and the firings of the pool of motor units that form a muscle (15, 21, 23). This relationship has been experimentally demonstrated before by the existence of EEG-EMG coherence at the tremor frequency (2, 3, 6–13, 15, 16, 24).

In our method, motor unit spike trains are identified from non-invasively recorded multichannel surface EMG recordings using the Convolution Kernel Compensation (CKC) technique (25–27). These firings are then used to construct the phase-locked estimator of the tremor-related activity in EEG. By using simulated data, we show that our method tracks reliably the tremor activity for a wide range of physiologically realistic conditions. We then apply this method to recordings from nine essential tremor (ET) and nine Parkinson's disease (PD) patients, and show that the method outperforms the traditional coherence approach when detecting tremor-related cortical activity.

## Multichannel EMG-driven identification of tremor-related activity

**Figure 1:**
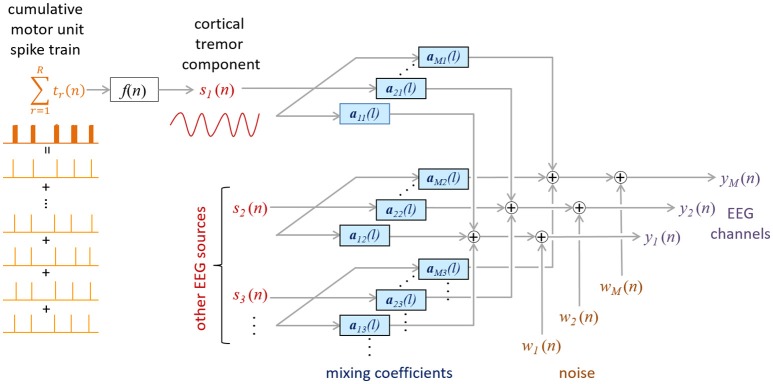
Proposed mixing model of the EEG. CST is coupled to the tremor EEG component *s*_1_(*n*) via an unknown function *f* (*n*). The other sources [*s*_2_(*n*) to *s*_*N*_(*n*)] represent the non-tremoric brain rhythms in the EEG. The impulse responses ***a***_*mj*_ project the *j*-th source *s*_j_(*n*) to the *m*-th EEG channel *y*_*m*_(*n*), whereas ω_*m*_(*n*) denotes the additive noise in the *m*-th EEG channel.

We first assume that EEG signals are linear mixtures of brain oscillations (rhythms) and noise (Figure 1). Then, using this data model, we build a linear minimum mean square error (LMMSE) estimator of the cortical tremor activity that exploits the large synchronization of motor unit firings in pathological tremor.

### Data model

Assume the mixing model depicted in Figure 1, and denote the *M* EEG channels by $y\left(n\right)={\left[y_{1}\left(n\right),y_{2}\left(n\right)\ldots y_{M}\left(n\right)\right]}^{T}$, where the *n*-th sample of the *i*-th channel appears in the *i*-th row of ***y***(*n*). The model inputs, *s*_*j*_(*n*), represent the brain rhythms in the EEG. For example, in a normal condition, these inputs would reflect the alpha, beta or gamma rhythms. As in other BSS/ICA EEG studies, artifacts, such as blinking artifacts, would also be considered a model input. In the case of pathological tremor, one or more of these inputs reflects tremor activity.

The mixing model in Figure 1 can be expressed in a matrix form as.

(1) $$y\left(\text{n}\right)=A\bar{s}\left(n\right)+\omega (\text{n})$$

Where

(2) $$\begin{array}{l} \bar{s}\left(n\right)=[s_{\text{1}}\left(n\right),s_{\text{1}}\left(n-1\right)\ldots s_{1}\left(n-L+1\right)\text{​},s_{\text{2}}\left(n\right)\ldots \\ \;{s_{2}\left(n-L+\text{1}\right)\ldots s_{J}(n-L+\text{1})]}^{T} \end{array}$$

contains blocks of *L* samples from *J* sources and the noise vector $\omega \left(n\right)={\left[\omega_{1}\left(n\right),\omega_{2}\left(n\right)\ldots \omega_{M}\left(n\right)\right]}^{T}\;$is modeled as a zero-mean ergodic Gaussian process, spatially and temporarily independent of the activity in **s**(*n*). The mixing matrix

(3) $$A=\left[\begin{array}{c} a_{11}\cdots \;a_{1J}\\ \vdots \;\ddots \;\vdots \\ a_{M1}\;\cdots \;a_{MJ} \end{array}\right]$$

contains stationary impulse responses **a**_*mj*_ = [*a*_*mj*_(0) ⋯ *a*_*mj*_(*L* − 1)] that convolve the *j*-th input source and add the result to the *m*-th EEG channel.

The model described in Equation (1) is typically underdetermined, with more inputs than measurements. However, low energy inputs can always be modeled as physiological noise and, thus, included in *ω*(*n*). We can also extend **y**(*n*) by adding *F-1* delayed repetitions of each EEG channel:

(4) $$\begin{array}{l} \bar{y}\left(n\right)=[y_{1}\left(n\right)\text{​},y_{1}\left(n-1\right)\ldots y_{1}\left(n-F+1\right)\text{​},y_{2}\left(n\right)\ldots \\ \;{y_{2}\left(n-F+1\right)\ldots y_{M}(n-F+1)]}^{T} \end{array}$$

This increases the number of outputs in model (1) and, more importantly, compensates potentially different time delays of same source in different EEG channels (see EMG-Driven Decomposition of EEG section for details). This is important because the same rhythm may be present at different EEG electrodes at close but different time lags (this assumption is further confirmed by the results in Results section). The extended input vector $\bar{\text{s}}$ and mixing matrix ***A*** now change to:

(5) $$\begin{array}{l} \bar{s}\left(n\right)=[s_{1}\left(n\right)\ldots s_{1}\left(n-L-F+2\right)\text{​},s_{2}\left(n\right)\ldots \\ {\;s_{2}\left(n-L-F+2\right)\ldots s_{J}(n-L-F+2)]}^{T} \end{array}$$

(6) $$\bar{A}=\left[\begin{array}{c} A_{11}\;\cdots \;A_{1J}\\ \vdots \;\ddots \;\vdots \\ A_{M1}\;\cdots \;A_{MJ} \end{array}\right]$$

with

$$A_{mj}=\left[\begin{array}{c} a_{mj}\;\cdots \;0\\ \vdots \;\ddots \;\vdots \\ 0\;\cdots \;a_{mj} \end{array}\right]$$

For the purpose of mathematical derivations in **Appendix**, we will further represent the EEG recordings **y**(*n*) as analytic signals. Note that this does not alter the assumed mixing model, and can be readily fulfilled by applying Hilbert transform to the EEG signals.

### EMG-driven decomposition of EEG

By temporarily neglecting the impact of noise *ω*(*n*), the LMMSE estimator of the input *s*_*j*_(*n*) is given by (25)

(7) $$\begin{array}{l} {\hat{s}}_{j}\left(n\right)=c_{s_{j}\bar{y}}^{H}C_{\bar{y}}^{-1}\bar{y}\left(n\right)=c_{s_{j}\bar{s}}^{H}{\bar{A}}^{H}{\left(\bar{A}C_{\bar{s}}{\bar{A}}^{H}\right)}^{-1}\bar{A}\bar{s}\left(n\right)\\ \;=c_{s_{j}\bar{s}}^{H}C_{\bar{s}}^{-1}\bar{s}\left(n\right), \end{array}$$

where superscript ^*H*^ denotes conjugate transpose, $c_{s_{j}\bar{\text{y}}}=E\left(s_{j}\left(n\right)\text{y}\left(n\right)\right)$ is the cross-correlation vector between *s*_*j*_(*n*) and **y**(*n*), and $c_{s_{j}\bar{\text{s}}}=E\left(s_{j}\left(n\right)\bar{\text{s}}\left(n\right)\right)$ is the cross-correlation vector between *s*_*j*_(*n*) and $\bar{\text{s}}\left(n\right)$. Matrices $C_{\bar{\text{s}}}$ and $C_{\bar{\text{y}}}$ denote the correlation matrices of $\bar{\text{s}}\left(n\right)$ and $\bar{\text{y}}\left(n\right)$, respectively. This LMMSE estimator is Bayesian optimal in the minimum square error sense, also in the presence of noise, but requires a priori knowledge of the cross-correlation vector $c_{s_{j}\bar{\text{y}}}$. In experimental conditions $c_{s_{j}\bar{\text{y}}}$ is not known and needs to be estimated from the measurements.

To obtain an estimate of ${\text{c}}_{s_{j}\bar{\text{y}}}$, we developed a method that is based on the assumption that, in an affected muscle, the motor unit firings are phase-locked to the tremor-related cortical activity. This assumption follows from the observations of significant coherence (linear relationship) between the EEG and the rectified EMG (1–10, 13, 14, 16) or, more directly, between the EEG and the motor unit CST (15). Remarkably, the number of motor units needed to represent population behavior during tremor is not very large, which in practice will favor the implementation of the proposed method. For example, in Negro and Farina (21) and Gallego et al. (15), the coherence between the CST and the EEG was nearly maximal with only five motor units and did not increase significantly when more motor units were added to the CST.

To obtain an estimate of $c_{s_{j}\bar{\text{y}}}$, we first define the firing pattern of the *r*-th tremor-affected motor unit:

(8) $$t_{r}\left(n\right)=\sum_{k=0}^{K_{r}}\delta \left(n-k\frac{f_{s}}{f_{r}}-d_{r}-\Delta d_{rk}\right),\;r=1,...,R,$$

where δ(τ) denotes the unit-sample impulse, *d*_*r*_ is the common time lag (in samples) of the pulses in *t*_*r*_(*n*) due to the transmission from motor cortex, Δ*d*_*rk*_ is the intrinsic motor unit firing time variability (in samples), and is frequently defined as the *k*-th realization of Gaussian random variable Δ*d*_*rk*_ ~ *N*(0, σ_Δ_*d*__*r*__), *f*_*r*_ is the motor unit firing frequency, *f*_s_ is the sampling frequency and *K*_*r*_ is the number of firings in the observed time interval. Note that, in order to simplify analytical derivations, we ignored the doublets in the motor unit firing pattern (8). This has small impact on the results presented herein, as doublets can always be modeled as two different instances of the *k*-th motor unit firing with different Δ*d*_*rk*_ values. Furthermore, as shown in Results section, when respecting its physiologically induced range, Δ*d*_*rk*_ has almost negligible impact on the proposed tremor estimation.

The cross-correlation between the EEG component *s*_*j*_(*n*) and **y**(*n*) can be estimated as (see derivation in the **Appendix**)

(9) $${\hat{c}}_{s_{j}\bar{y}}\approx \frac{\alpha }{N}\sum_{r=1}^{R}\sum_{\eta =1}^{N}t_{r}\left(\eta \right)\bar{y}(\eta )$$

where *N* is the number of signal samples and factor α is defined in the **Appendix**). Due to the amplitude ambiguity of source components extracted by BSS (28), the scalar factor α can be neglected and the EEG-component that is phased-locked to the firings of the *J* motor units expressed as

(10) $${\hat{s}}_{j}\left(n\right)={\hat{c}}_{s_{j}\bar{y}}^{H}C_{\bar{y}}^{-1}\bar{y}\left(n\right)$$

By knowing ŝ_*j*_(*n*) cross-correlation vector ${\hat{\text{c}}}_{s_{j}\bar{\text{y}}}$ in (9) may be recalculated as

(11) $${\hat{c}}_{s_{j}\bar{y}}\approx {ℱ}^{-1}\left(g\left({\hat{S}}_{j}\left(f\right)\right)\right)\bar{y}(\eta )$$

where ${\hat{S}}_{j}\left(f\right)=\;F\left({\hat{s}}_{j}\left(n\right)\right)$ is Fourier transform of ŝ_*j*_(*n*) and *g*(*x*) = x*|*x*| denotes the element-wise product of element x with its absolute value. Equations (10, 11) are then iteratively recalculated until the convergence is reached. In our study, iterations were stopped when the second norm of ŝ_*j*_(*n*) changed for < 0.1%. These iterations emphasize the spectral peaks in the extracted EEG component and suppress the wideband frequency components with low energies.

Note that (9) is only true when none of the other oscillatory inputs in ***s***(*n*) is a higher harmonic of the input *s*_*j*_(*n*). In the opposite case, (10) would identify both the tremor-related EEG component and its higher harmonics as one joint input (see explanations in the **Appendix**).

The presented method still needs a good approximation of the motor unit firing times, ${\hat{t}}_{r}\left(n\right)$, in order to accurately estimate ${\hat{\text{c}}}_{s_{j}\bar{\text{y}}}$. This can be obtained from high-density surface EMG recordings using the CKC decomposition technique (25, 27, 29–31), which has already been demonstrated to be highly robust to high levels of motor unit synchronization (27).

## Simulations and experimental recordings

The presented method was validated on a set of synthetic signals and on experimental recordings from 18 tremor-affected patients.

### Simulations

First, we tested the proposed method in a simple model that generated EEG-like oscillations as mixtures of sinusoidal sources with time-varying amplitudes. Our goal was to test the method's ability to accurately reconstruct such EEG-like sources from their convolutive mixtures, and to study its sensitivity to its three main parameters: extension factor *F* in (4), motor unit firing variability Δ*d*_*rk*_ and signal-to-noise ratio (SNR).

The simulated signals comprised 10 (*J* = 10) mutually orthogonal sinusoids *s*_*j*_(*n*) and their first higher harmonics as input signals:

(12) $$s_{j}\left(n\right)=\text{a}(\text{n})\cdot \left(B\cdot \sin\left(2\pi f_{j}n-\phi_{j}\right)+H_{1}\cdot \sin\left(4\pi f_{j}n-\phi_{j}\right)\right),$$

where a(n) is an amplitude modulator generated by filtering white noise with a second order low-pass Butterworth filter with cut-off frequency of 1 Hz. The amplitude B was set equal to 1, whereas the amplitude of the first harmonic, *H*_1_, was varied across simulations and was set to 0, 0.2, 0.4, 0.6, 0.8, and 1. This way we simulated the experimentally observed ratios between the basic tremor frequency and its first harmonic (9, 13, 32). The frequency *f*_*j*_ of the oscillatory inputs was set to 5+*j*/2 Hz with *j* = 1, 2 … 10, and the phase ϕ_*j*_ was randomly selected from the interval [0, 2π]. The sampling frequency was set to 1024 Hz and each simulation lasted 30 s. We assumed that the first oscillatory input, *s*_1_(*n*), represented the tremor-related component we wanted to detect.

Next, we simulated the spike trains of ten motor units, *t*_*r*_(*n*) with *r* = 1, 2 … 10, by finding the local maxima of the first generated oscillatory source, *s*_1_(*n*). We imposed a corticospinal delay *d*_*r*_ = 10 ms in Equation (8) to simulate the physiological delays (due the transmission from motor cortex to the output of the motoneuron pool) between the tremor-related EEG component *s*_1_(*n*) and the simulated motor unit firing patterns *t*_*r*_(*n*). Finally, the firing variability Δ*d*_*rk*_ of each individual motor unit *t*_*r*_(*n*) was modeled as Gaussian random variable Δ*d*_*rk*_ ~ *N*(0, σ_Δ_*d*__*r*__) (33, 34) (see **Appendix**). The standard deviation σ_Δ_*d*__*r*__ was set to 0, 10, and 20% of the average inter-spike interval in *t*_*r*_(*n*), respectively (35–38). Ten Monte Carlo simulation runs were performed for each value of *H*_1_ and σ_Δ_*d*__*r*__, resulting in a total of 180 (10 × 6 × 3) simulation runs.

We computed 15 synthetic channels **y**(*n*) in Equation (1), with the mixing matrix **A** having dimensions 15 × 50. The length of the impulse responses **a**_*mj*_(*l*) in (3) was set to *L* = 5 samples. To simulate different delays in the representation of the oscillatory inputs *s*_*j*_(*n*) across the 15 channels, each element **a**_*mj*_ had one randomly selected element set to a non-zero random value from a normal distribution *N*(0, 1), whereas the remaining four elements were set to zero. In each simulation run, five different realizations of zero-mean random Gaussian noise were added to the measurements, with the SNR ranging from 0 to 20 dB in steps of 5 dB. This resulted in 900 (180 × 5) simulated sets of signals. Note that our simple generative model does not realistically represent actual EEG signals; our goal was to use the simulation results to identify the range of parameter values that was adequate for the experimental data analysis. All the simulations were performed in Matlab version 8.6.0.267246 (R2015b).

### Experimental recordings

We recorded data from nine ET patients (four females and five males; age, mean ± SD: 70 ± 6 years, range 61–79 years) and nine PD patients (three females and six males; age, mean ± SD: 64 ± 14 years, range 44–88 years) at Hospital 12 de Octubre, Madrid, Spain. In the ET patients, tremor severity ranged from mild (two patients) to severe (three patients), with a mean score of 36 ± 12 (mean ± SD; range 20–51) according to the Fahn-Tolosa-Marin scale (39). In the PD patients, tremor severity also ranged from mild (five patients) to severe (two patients), with a mean score of 12 ± 6 (mean ± SD; range 5–23) according to the UPDRSIII scale (40). All the participants included in the study gave their written informed consent after full explanation of the procedure. The study, which was conducted in accordance with the principles of the Helsinki declaration of 1975, was approved by the ethical standards committee on human experimentation at the University Hospital “12 de Octubre” (Madrid).

The experimental protocol comprised three repetitions of a 30 s long postural task, during which patients kept their arms outstretched, parallel to the ground for 30 s. During these tasks, we measured EEG, multichannel surface EMG, and wrist kinematics. EEG was recorded with 32 passive Au or active Ag/AgCl electrodes (depending on the session) placed on a cap that fulfilled the extended 10/20 system (g.Tec GmbH, Graz, Austria). Electrodes were placed in the following positions: AFz, F3, F1, Fz, F2, F4, FC5, FC3, FC1, FCz, FC2, FC4, FC6, C5, C3, C1, Cz, C2, C4, C6, CP5, CP3, CP1, CPz, CP2, CP4, CP6, P3, P1, Pz, P2, and P4. The ground was placed on the AFz position, with linked earlobes used as a reference. Before positioning the electrodes the skin was slightly abraded with abrasive paste (Meditec–Every, Parma, Italy) and conductive gel (Meditec–Every, Parma, Italy) was put under the electrodes. The recorded signals were amplified (gUSBAmp, g.Tech GmbH, Graz, Austria), band–pass (0.5–60 Hz) and notch filtered (50 Hz), to remove power line interference, and sampled at 256 Hz with 24 bit resolution.

Right wrist kinematics were recorded with inertial measurement units (IMUs) comprising three-axis accelerometers, magnetometers and gyroscopes (Tech MCS, Technaid S.L., Madrid, Spain). These sensors were fixed with surgical tape over the hand dorsum and the distal third of the forearm (on the dorsal side, close to the wrist), respectively, with one of their axes aligned to that of the wrist. Data were sampled at 100 Hz by a 12-bit A/D converter and low pass filtered (< 20 Hz). Wrist kinematics was assessed as the difference between the measured accelerations in the axis parallel to the wrist (41).

Surface EMG signals were recorded from the right wrist flexors and extensors with 13 × 5 electrode grids (LISiN–OT Bioelettronica, Torino, Italy, 8 mm interelectrode distance). The electrode grids were centered over flexor carpi radialis and extensor digitorum communis, respectively. Before the placement of the electrode grid, the skin was lightly abraded using abrasive paste (Meditec–Every, Parma, Italy) and cleansed afterward. Electrical conductivity was ensured by filling each of the electrodes in the grids with conductive gel (Meditec–Every, Parma, Italy). A soaked bracelet placed around one of the wrists was used as reference. The surface EMG signals were amplified as bipolar recordings along the direction of the fibers, band-pass filtered (3 dB bandwidth, 10–750 Hz), and sampled at 2,048 Hz by 12–bit A/D converter (LISiN–OT Bioelettronica, Torino, Italy). We synchronized the EEG, EMG, and IMU recordings using a common clock signal, which was fed into each acquisition systems. The rising edge of the first and last clock signal pulses were identified using a purposely-developed Matlab script. Data were then resampled to 2,048 Hz using a routine that incorporated an anti-aliasing filter.

### Data analysis

In the experimental recordings, individual motor unit firing patterns were identified from the multichannel surface EMG using the CKC algorithm (25, 27, 29). The pulse-to-noise ratio metric (PNR) (42) was used to assess the accuracy of firing estimation for each identified motor unit. Only reliably identified motor units (PNR > 30 dB; accuracy of motor unit firing estimation >90%) were used for further analysis (42), whereas all the remaining motor units were discarded.

We estimated the tremor EEG component with Equation (7), using the firings of all the identified (experimental recordings) or simulated (simulations) motor units to estimate the cross-correlation ${\hat{\text{c}}}_{s_{j}\bar{\text{y}}}$ between the oscillatory components and the EEG signals, as defined in Equation (10). We tested different extension factors, from *F* = *1* to *F* = *15*. Due to the amplitude ambiguity (see **Appendix**), the estimate ŝ_1_(*n*) was further normalized to yield a unit norm. Finally, Equations (10, 11) were iteratively applied until the convergence criterion was reached (the second norm of ŝ_*j*_(*n*) changed for < 0.1%).

The delay between the motor unit CST and the estimated tremor EEG component ŝ_1_(*n*) was estimated as the argument of the cross spectrum at the basic tremor frequency *f*_*b*_ (17, 43).

In the simulations, we further assessed the accuracy of our method by computing the normalized mean square error (NMSE; Equation 13) and the cross-correlation coefficient (CC) between the simulated and estimated tremor inputs, ŝ_1_(*n*) and *s*_1_(*n*), after both signals were aligned in time:

(13) $$NMSE=\frac{\sum_{n=1}^{N}{\left(s_{1}\left(n\right)-{\hat{s}}_{1}\left(n\right)\right)}^{2}}{\sum_{n=1}^{N}{\left(s_{1}\left(n\right)\right)}^{2}}\cdot 100$$

In the experimental data, we compared the coherence between the extracted tremor component and the CST in each muscle to the coherence between the CST and the Laplacian-filtered EEG (15) (without any artifact rejection applied). In all these cases the CST was smoothed by convolving it with a 25 ms long Gaussian window. The 99% confidence limit of the coherence function was obtained as (17):

(14) $$\text{1}-{\left(\text{0}.\text{01}\right)}^{\text{1}/(L-\text{1})}$$

where *L* is the number of disjoint 1-s segments used in the spectral estimation.

Finally, we computed the relative power H1/(H1+B) of the first tremor harmonic with respect to the basic tremor frequency in the estimated tremor EEG component, ŝ_1_(*n*).

Due to their non-Gaussian distribution (Lilliefors test, *p* > 0.05), the non-parametric Kruskal–Wallis test was used to compare the differences between the ET and PD patient groups, whereas the Wilcoxon signed rank test was used for paired comparisons. The significance level was set to *p* < 0.05 and *p* < 0.01, respectively (see Results section for details).

## Results

### Simulated data analysis

**Figure 2:**
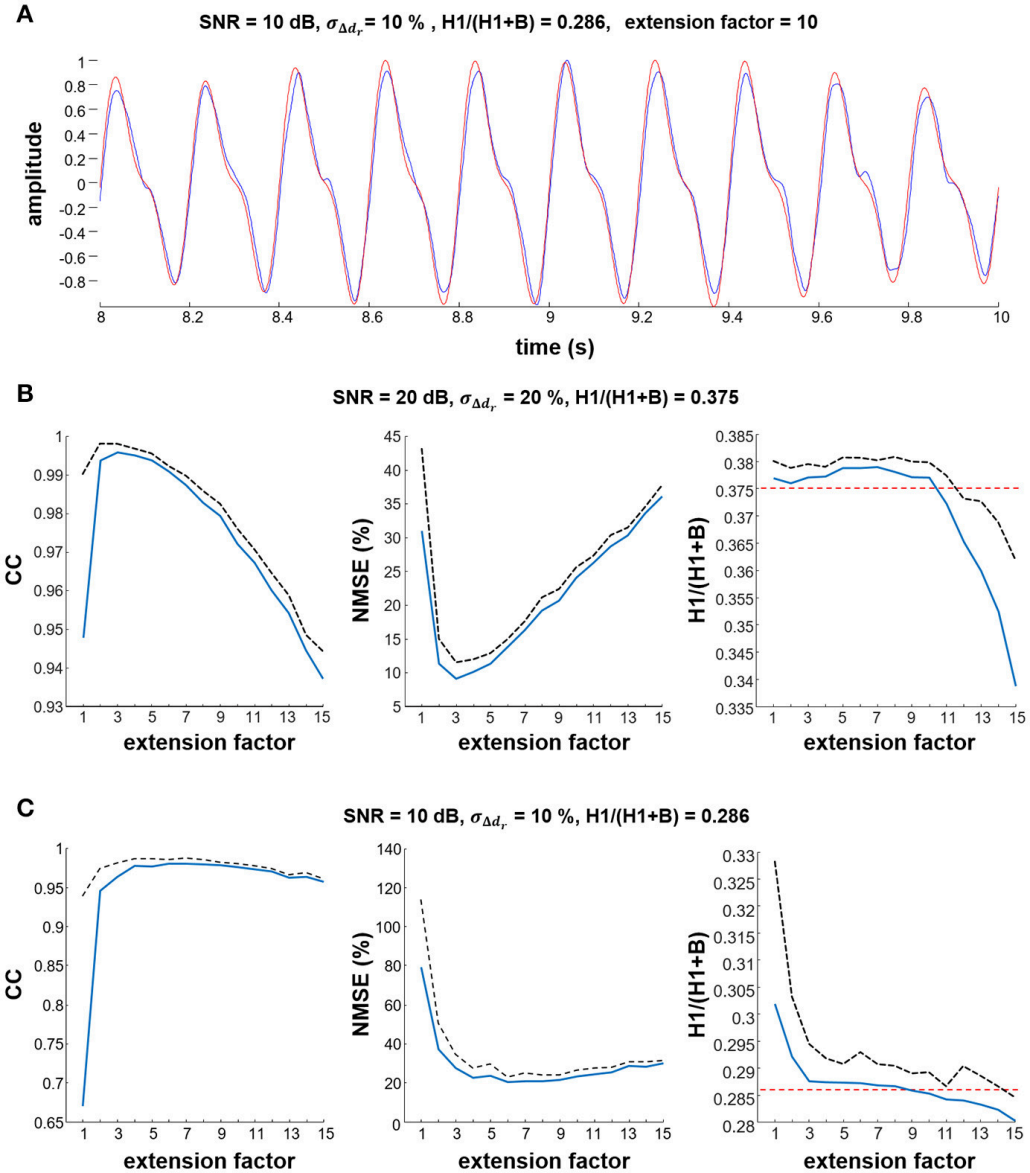
Estimation of the simulated tremor component. **(A)** Representative example showing that the estimated tremor component (blue line) was similar to the simulated source (red line). In this example, simulation parameters were SNR = 10 dB and **σ**_**Δ**_***d***__***r***__ = 10%. **(B,C)** impact of different values of the extension factor *F* on the estimated cross-correlation coefficient (CC), the NMSE between estimated and reference tremor component, and the H1/(H1+B) ratio. Results are averaged over 10 simulation runs. Mean values are depicted as thick blue lines, standard deviations as black dashed lines. In the H1/(H1+B) ratio plots, the simulated reference values are depicted with red dashed lines.

Figure 2A shows a representative example of detection of the tremor component in the simulated signals, demonstrating that the proposed method accurately extracts the tremor-related component from the synthetic signals. Figures 2B,C show the correlation between the estimated and simulated sources, the NMSE and the estimated H1/(H1+B) ratio as a function of extension factor *F*, for two different SNR and σ_Δ_*d*__*r*__ values. Note the high correlation and small NMSE between the simulated and the estimated tremor-related component, and the accuracy with which the ratio H1/(H1+B) was detected when extension factor was set to *F* = 3 or higher. For a SNR of 20 dB, extension factors from *F* = *2* to *F* = *5* were optimal, whereas for lower SNRs, extension factors from *F* = *4* to *F* = *9* were optimal (Figure 2C). In both cases the estimated H1/(H1+B) ratio was largely independent from the extension factor in the interval *F* = *3* to *F* = *10*. All metrics degraded slightly when the model looked too many samples back in the past (*F* > 10). Based on these results and on the coherence analysis of experimental data (Figure 6), we selected an extension factor *F* = 8 for further analyses. Note that Figure 6 indicates that our results would hold across a broad range of values of *F*, from *F* = 5 to *F* = 10.

**Figure 3:**
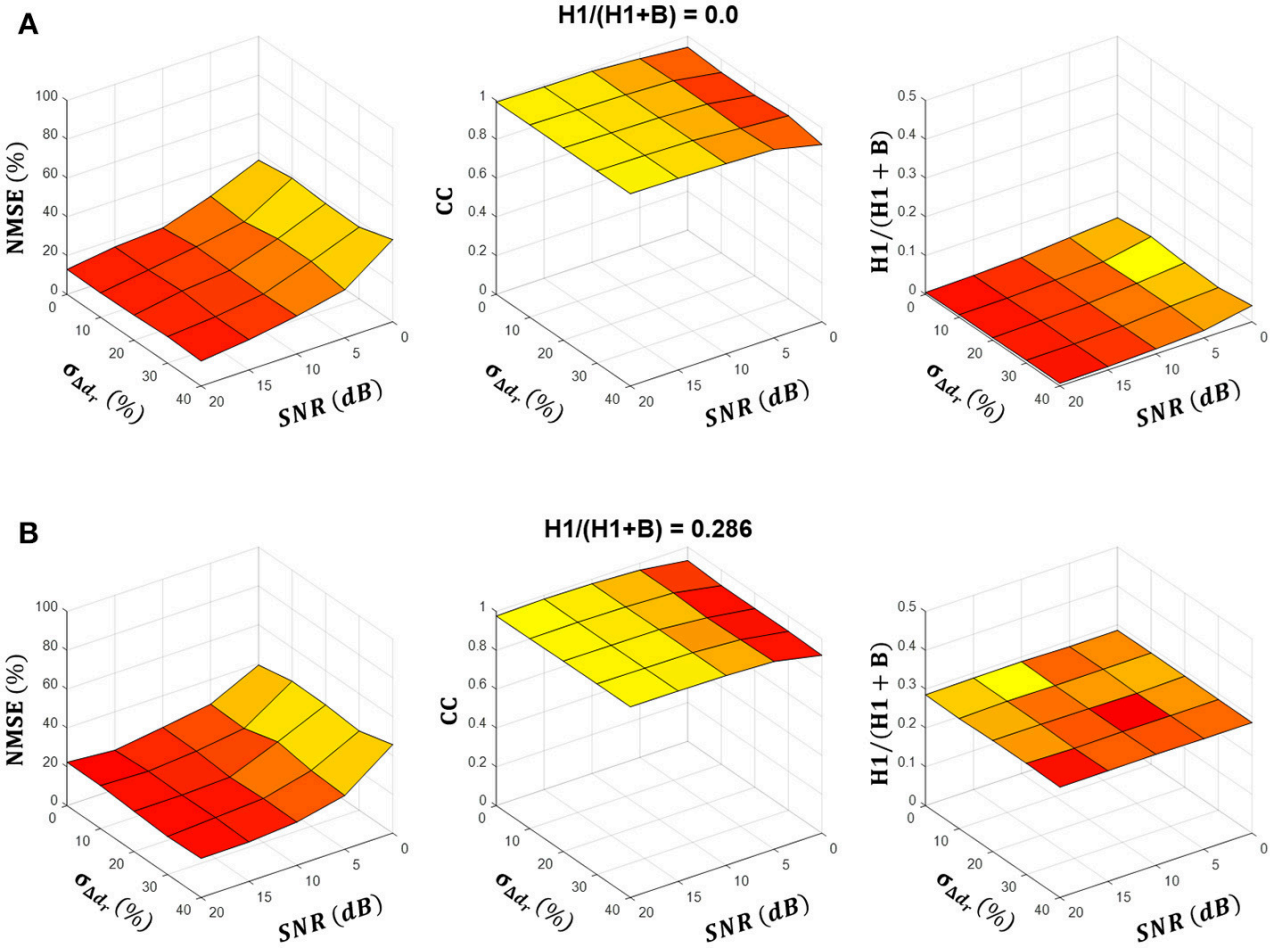
Summary results of estimation of the tremor component across simulated conditions. **(A,B)** show the NMSE and the cross-correlation (CC) between the estimated and simulated tremor source, and the estimated H1/(H1+B) ratio as a function of parameters SNR and **σ**_**Δ**_***d***__***r***__ for simulated H1/(H1+B) ratios of 0.0 and 0.286, respectively. Results are averaged over ten simulation runs and depicted as a mean (colored surfaces) and mean + SD (black lines). Note that SD was negligibly small.

Figures 3A,B summarize the NMSE, CC and H1/(H1+B) ratio as functions of the SNR and motor unit firing time variability σ_Δ_*d*__*r*__ at two different simulated H1/(H1+B) ratios. In both cases, the NMSE decreased and the CC increased as the SNR increased, whereas they did not change significantly with σ_Δ_*d*__*j*__, which suggests that the simulated intrinsic variability in motor unit firing did not affect the source estimation. The estimated H1/(H1+B) ratio did not change significantly with the SNR or σ_Δ_*d*__*r*__ and was always very close to the simulated H1/(H1+B) ratio. Namely, when averaged over all simulated SNRs, σ_Δ_*d*__*r*__ and H1/(H1+B) ratios, the difference between the simulated and estimated H1/(H1+B) ratio was 0.01 ± 0.05.

The delay between the estimated and simulated sources, ŝ_*j*_(*n*) and *s*_*j*_(*n*) was largely independent of the SNR, σ_Δ_*d*__*r*__, and the simulated H1/(H1+B) ratios, averaging 0.4 ± 1.4 ms across all combinations of parameters. When a 10 ms corticospinal delay was imposed between the motor unit firings and their cortical drive *s*_*j*_(*n*), the estimated delay averaged 11.0 ± 1.6 ms. This implies a 1.0 ± 1.6 ms difference with the simulated 10 ms delay. Despite this estimate being quite accurate, we want to note that the current simulations do not generate signals as complex as the recorded EEGs. Nor did the simulations incorporate the delays due to propagation of the motor unit action potentials along the muscle fibers or due to EMG decomposition with CKC (25, 26, 27). All these factors contribute to the unknown global delay between 0 and ~15 ms and cannot be easily estimated in experimental conditions. Thus, we do not expect the experimental estimates of corticospinal delay to be as accurate as those obtained based on the model.

### Experimental recordings

**Figure 4:**
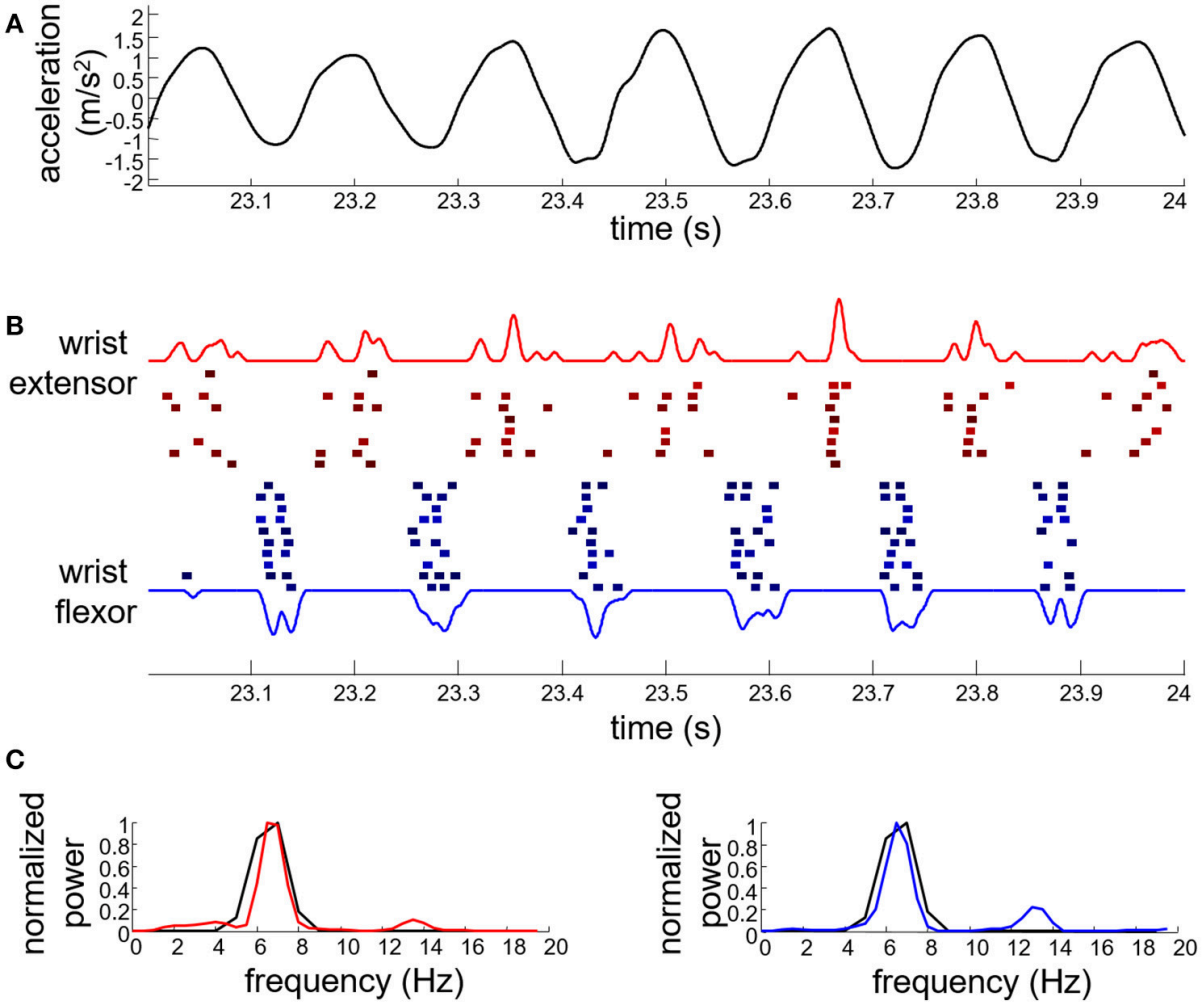
Example of tremor data from an essential tremor patient performing the postural task. **(A)** Wrist acceleration; **(B)** identified motor unit firings for the wrist extensors (red squares) and flexors (blue squares) along with the corresponding smoothed CST (red and blue solid lines; the blue trace is inverted for representation purposes). Each square denotes a single motor unit firing. Firings of different motor units are depicted with different vertical offset; **(C)** power spectrum of the smoothed CST (red solid line for wrist extensors and blue solid line for wrist flexors) compared to that of the wrist acceleration data (black solid line).

Figure 4 shows an example of EMG decomposition in a representative ET patient, along with the smoothed CST. Table 1 summarizes the number of motor units that were detected from the surface EMG with PNR > 30 dB and then used for the identification of the tremor EEG component. On average, 7.7 ± 5.2 and 8.6 ± 6.3 motor units per contraction were identified for the ET and PD patients, respectively. The average number of firings per motor unit was 160 ± 100 for the ET and 218 ± 130 for the PD patients (Table 1). In each contraction, all the accurately identified motor units per muscle were used to estimate the tremor EEG component (see Appendix). Since we recorded EMGs from the wrist extensors and flexors of the right arm, two estimates of tremor EEG components were extracted per each task repetition.

**Table 1:** Summary of the properties of the motor units, identified by multichannel EMG decomposition.

		**EXT R**	**FLE R**
ETs	No. MUs	9.0 ± 5.2 (2–23)	5.4 ± 2.8 (2–13)
	No. firings	200 ± 108 (37–531)	107 ± 72 (36–341)
	PNR (dB)	35.0 ± 4.3 (30.1–51.2)	33.0 ± 4.2 (30.1–51.2)
PDs	No. MUs	9.8 ± 6.1 (1–23)	6.0 ± 5.0 (1–33)
	No. firings	255 ± 126 (36–502)	147 ± 108 (36–414)
	PNR (dB)	34.2 ± 4.0 (30.1–50.5)	34.7 ±4.2 (30.1–49.8)

*The table shows the number of identified motor units with PNR > 30 dB, their number of firings, and the PNR, for right wrist extensors (EXT R) and flexors (FLE R) of 9 the ET and 9 PD patients. Results are reported as mean ± SD (range)*.

**Figure 5:**
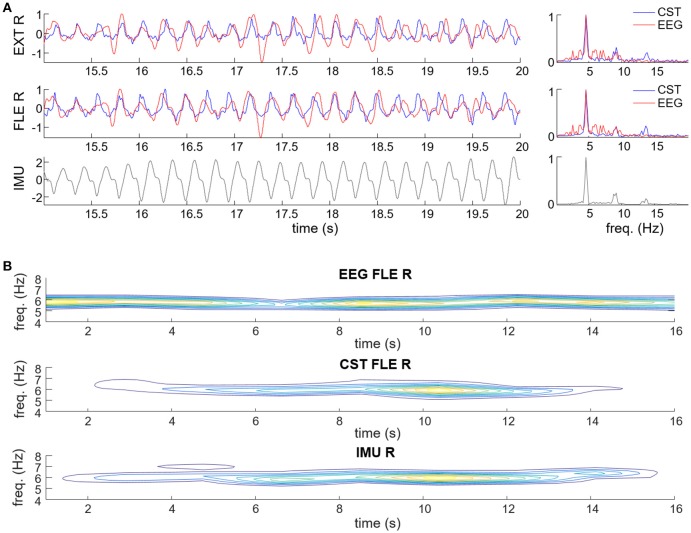
Examples of estimation of the tremor-related cortical activity in experimental recordings of a representative PD **(A)** and ET patient **(B)**. **(A)** Estimated tremor EEG component (red traces) compared to smoothed CST (blue traces) of the right wrist flexor (FLE R) and extensor muscles (EXT R), and wrist acceleration (IMU; displayed in black). Data are plotted in time (left plots) and frequency domain (right plots). **(B)** Contour plots of the spectrograms of the extracted tremor-related EEG component, smoothed CST of right wrist flexor (CST FLE R) and right wrist acceleration. Warmer colors represent higher power.

Figure 5 shows a representative example of the estimated tremor EEG component. Figure 5A depicts the estimated tremor EEG component and how it relates to the smoothed CST, both in the time (left plots) and the frequency domain (right plot). The estimated EEG component exhibits clear tremor-related activity with peaks both at the basic frequency and the first harmonic of that observed in the pooled motor unit firings and the wrist kinematics. Figure 5B shows time-frequency domain contour plots of the extracted EEG component, the smoothed CST and the wrist kinematics, as reference. In the presented case, the tremor-related EEG activity preceded the pooled motor unit activity and the observed wrist movements, which manifested simultaneously, but this was not always the case.

**Figure 6:**
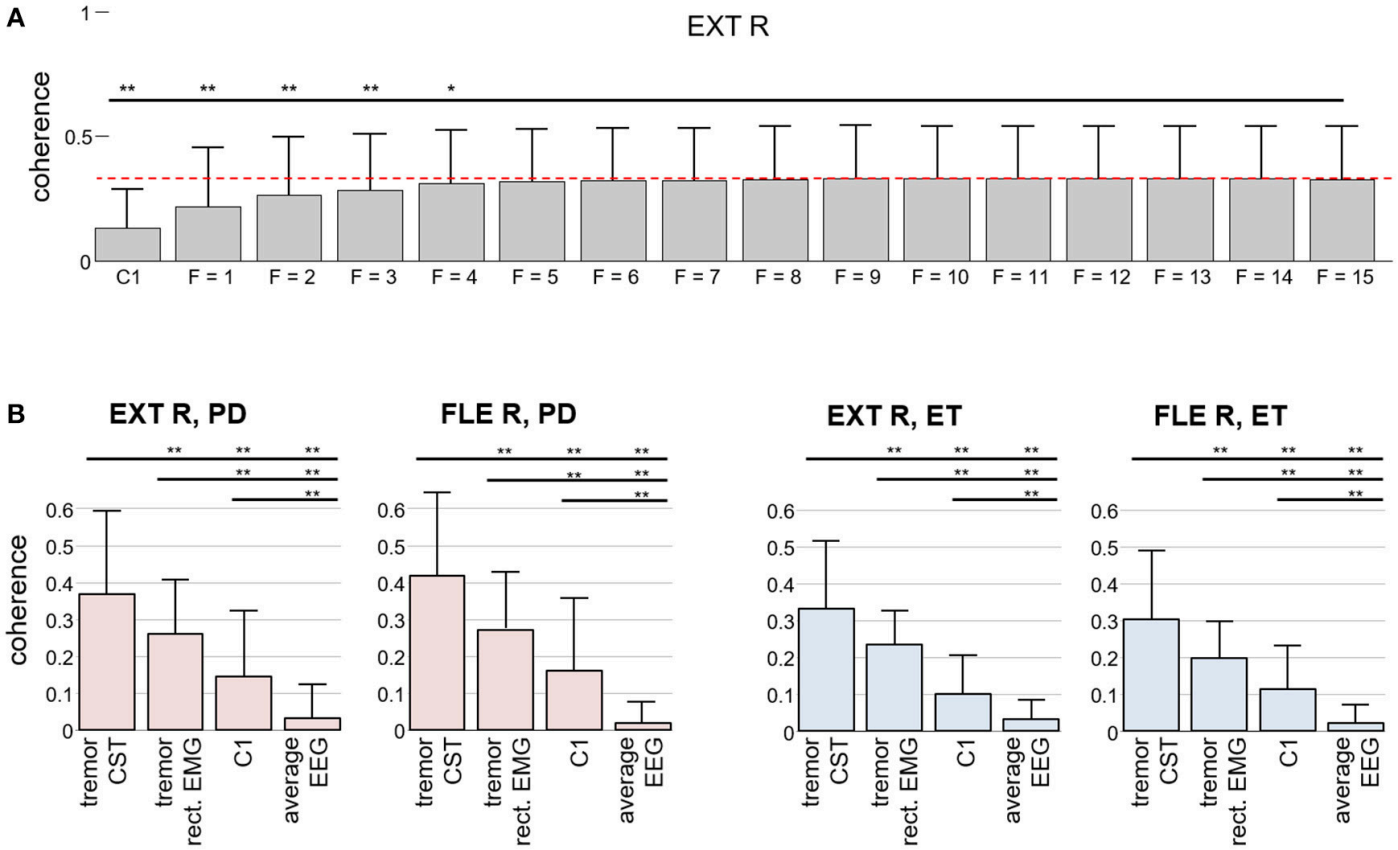
Comparison of our method for detecting tremor-related cortical activity to the traditional coherence approach in experimental recordings. **(A)** Coherence between the CST of the right wrist extensor and the estimated tremor EEG component as a function of the extension factor *F*. For reference, the average coherence with the EEG signals over C1 is also depicted. The results are averaged over all the trials from the PD and ET patients and reported as mean (bars) and SD (whiskers). **(B)** Coherence between the Laplacian-filtered EEG and the CST in comparison with the coherence between the extracted tremor EEG component and the CST (our proposed method, *F* = 8): Two results for CST-EEG coherence are depicted in each graph, one for coherence between CST and spatially averaged EEG (“average EEG”) and one for coherence between CST and EEG channel at C1 position (“C1”). Similarly, two results for coherence between CST and extracted tremor component are depicted, one for tremor component, extracted from EEG by using the CST-based estimation described in the Appendix (“tremor CST”) and one for tremor component, extracted from EEG by replacing the CST in Appendix by the spatially averaged rectified EMG, low pass filtered at 15 Hz (“tremor rect. EMG”). Results are plotted separately for each investigated muscle. Superscripts *p < 0.05 and **p < 0.01 denote statistically significant difference as assessed by Wilcoxon signed rank test.

We investigated how the number of EEG samples in the time domain (extension factor *F*) influenced the accuracy of the estimation of the tremor-related cortical activity. To this end, we computed the coherence between the tremor EEG component and the smoothed CST for increasing values of *F* (from *F* = 1 to *F* = 15). As shown in Figure 6A, the coherence first increased, but it saturated around extension factor *F* = *8*, in agreement with the simulations with lower SNR ratios. Note, however, that the increase in coherence as more EEG samples were included was not significant after *F* = 5. Figure 6B demonstrates that the proposed method significantly outperforms the classical coherence between Laplacian-filtered EEG and spatially averaged rectified EMG, low pass filtered at 15 Hz.

**Figure 7:**
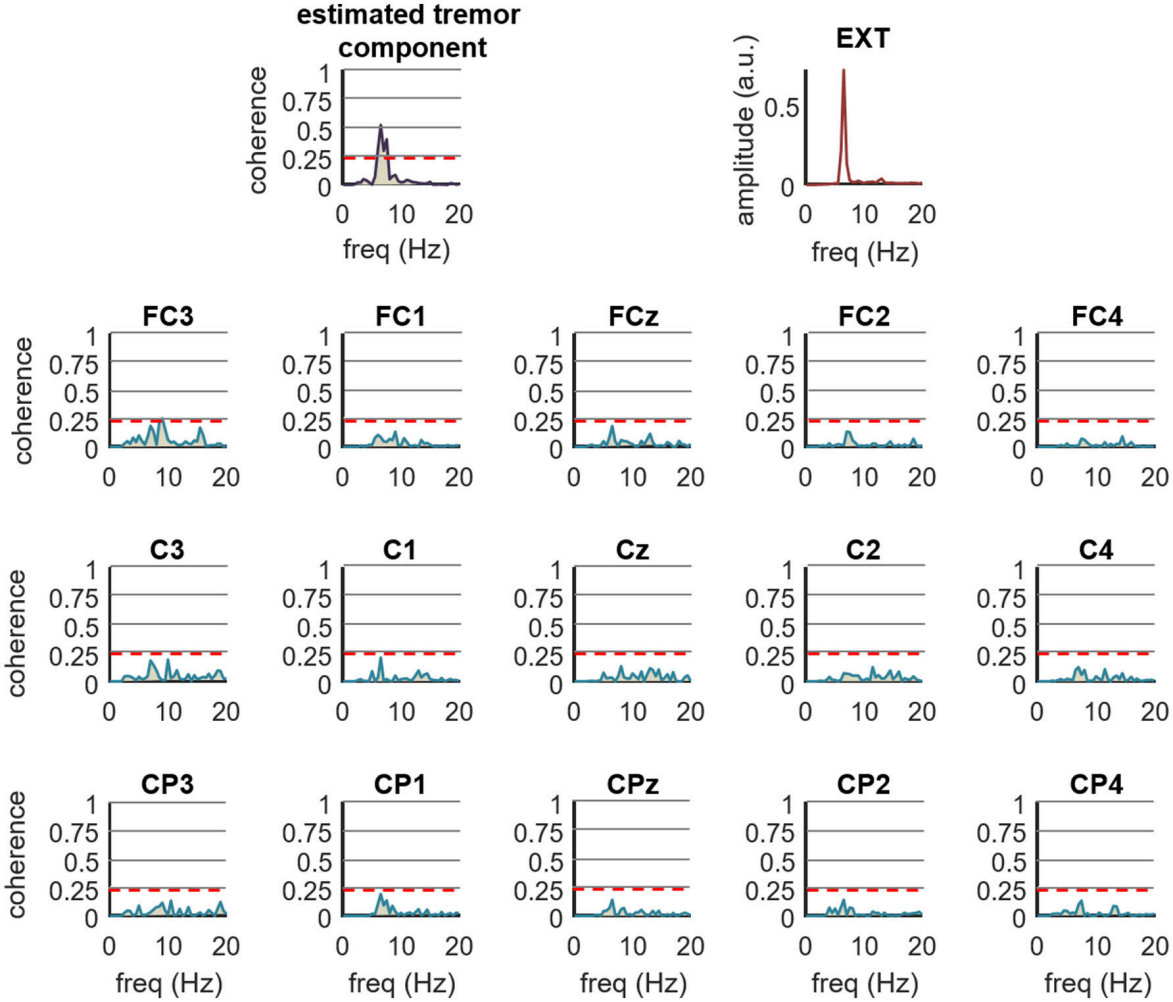
Comparison of the proposed method for the detection of tremor-related cortical activity (*F* = 8) with the traditional coherence approach in experimental recordings. The top right plot shows the spectrum of the smoothed CST form right wrist extensor of an ET patient performing the postural task (EXT). The top left plot shows the coherence between the CST and the tremor EEG component as estimated by the proposed method, while the remaining 12 plots show the coherence between the Laplacian filtered EEG signals and the CST. The label on top of each plot indicates the central EEG electrode. Red dashed lines represent the 99% confidence limit. Note the increase in coherence yielded by our method compared to the traditional coherence approach. Coherence calculated by traditional approach was not significant in any of the electrodes.

Figure 7 shows an example of how the proposed method performs compared to the traditional approach of computing the coherence between an estimate of muscle activity (in this case the smoothed CST) and the spatially filtered EEG signals. We calculated the coherence function for the entire recordings (1 s windows with 50% overlapping), and found no significant coherence values in any EEG channel. In contrast, the coherence between the CST and the EEG component extracted using the proposed method (extension factor: *F* = 8) was significant. These findings generalized to the all the tested ET and PD patients (Figure 6B). Our proposed method thus outperforms classic coherence approaches.

In 52% of cases studied (28 of 54), the tremor-related EEG component preceded the CST by 11.0 ± 6.4 ms, whereas in the remaining 48% of cases, the CST preceded the extracted EEG component by 11.0 ± 5.9 ms. All the delays were clustered on the interval between −30 ms and + 30 ms, and we observed no significant difference between the delays in PD and ET patients (*P* > 0.05, Kruskal–Wallis test). These latency values are in agreement with previous studies (7–10, 13), notwithstanding the limitation listed in the Discussion section.

**Figure 8:**
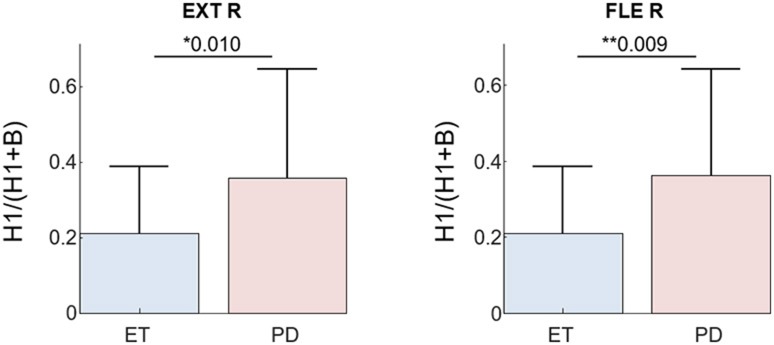
H1/(B+H1) ratio of the estimated tremor EEG component (*F* = *8*) for all PD and ET patients during the postural task. Results are plotted separately for each investigated muscle; Superscripts *p < 0.05 and **p < 0.01 denote statistically significant difference as assessed by Kruskal–Wallis test.

Finally, a significant difference was observed in the H1/(B+H1) ratio of the extracted tremor EEG between PD and ET patients (Figure 8), also in agreement with previous studies (9, 13).

## Discussion

In this study, we derived and validated a new method for the extraction of the tremor-related EEG activity in the case of pathological tremor. The method builds on the physiological coupling between the tremor-related cortical activity and the neural drive to the muscle (the output of the motoneurons that innervate a muscle). In particular, our method combines the motor unit spike trains identified in the decomposition of high-density surface EMG recordings to build an estimator of the tremor-related EEG component. We applied it to EEG recordings to demonstrate its feasibility, but it could also be used for analyzing magnetoencephalographic (MEG) data.

The proposed method was tested on simulated data and on recordings from 9 PD and 9 ET patients. In the simulations, our method detected the simulated tremor component with great accuracy, as indicated by the low NMSE and high cross-correlation values. The small difference between the simulated and estimated H1/(B+H1) ratio (Figures 2, 3, global average error of 0.006 ± 0.053 for simulated H1/B, ranging from 0 to 1) further demonstrates the fidelity of the estimated tremor component. Our method also yielded very accurate estimates of the delay between the motor unit population activity and the simulated EEG (the average error was 1.0 ± 1.6 ms for a simulated delay of 10 ms).

In the experimental data, the extracted tremor EEG component exhibited clear similarities with the recorded kinematics and motor unit population activity, both in the time and frequency domains. The ground truth about the estimated EEG tremor component is not available in experimental conditions. However, we believe our method performed well because the estimated EEG component exhibited significantly larger coherence with the identified population of motor units than the spatially filtered EEG signals, which is the standard approach (1–10, 13, 16). This observation indicates that the proposed method is likely to help studying the neural mechanisms of tremor. Indeed, our method always identified tremor-related activity in the EEG, while in many of the investigated cases (34 of 54) we did not find significant coherence between the spatially filtered EEG signals and the identified population of motor units. Note that this observation is in agreement with reports that several tens of second long recordings are needed to obtain robust results in standard coherence analysis (2, 6–10, 13), whereas our datasets were only 30 s long.

Movement artifacts are an important potential confound when studying corticospinal coupling using coherence techniques. We performed two complementary analyses to discard the presence of movement artifacts. First, we tested whether the tremor components were present across many spatially filtered EEG channels, as it would be the case if they resulted from movement artifacts. We calculated the coherence between the CST and each spatially filtered EEG channel. As reported above, in 34 out of 54 cases we did not find significant coherence between the spatially filtered EEG signals and the identified population of motor units. In the remaining 20 cases, significant coherence at the tremor frequency or at its higher harmonics was observed on one or two EEG channels only. As a second control, we examined whether the EEG-CST delays depended on the basic tremor frequency. Finding a significant association between these two parameters would indicate a potential mechanical coupling. Our results ruled out this possibility: the EEG-CST delays lied within the −30 to 30 ms interval. These values did not overlap with the range of delays potentially indicating an artifact (from ~45 to 100 ms; interval defined by the maximum and minimum tremor frequencies, 11 and 5 Hz, respectively). Therefore, our control analyses indicate that the identified tremor component is unlikely to originate from movement artifacts. Note that these extensive tests are necessary every time the presented methodology is used as, similar to the classical coherence analysis, the movement artifacts could completely mask any tremor-related activity in cortex.

The only parameter that needs to be chosen in our method is the extension factor *F* in Equation (4). In simulated conditions, the optimal extension factor was dependent on the SNR (of the input sources in the simulated EEG signals) with the optimal values between *F* = 2 and *F* = 5 for SNR of 20 dB, whereas larger values, between *F* = 4 and *F* = 9, proved to be optimal in the case of lower SNRs (Figure 2). We decided to choose extension factor *F* = 8 for subsequent analyses. This choice was confirmed during the analysis of the experimental signals, because the coherence between the estimated tremor-related EEG component and the smoothed CST reached the plateau region at *F* = *8*, whereas the overall increase in coherence was not significant after *F* = *5* (Figure 6A). The observation that in all the cases studied *F* = *1* yielded significantly worse results (lower coherence), indicates that the extension of the convolutive model (1) helps in coping with either the existence of different delays in the representation of brain rhythms across different EEG channels, or with the convolutive nature of EEG mixtures. Since the computational complexity of the proposed method increases with the square of the extension factor *F*, it is to our advantage that the preferred value of *F* is relatively small.

Regarding the neurophysiological results of this study, we found that the relative power of the first tremor harmonic compared to the basic tremor frequency is greater in PD than ET patients, regardless of the investigated muscle (Figure 8). This is in agreement with other studies using EEG-EMG coherence (9, 13). The observed delay between the estimated tremor EEG component and the pooled motor unit firings also agrees with previously reported values. Several studies in ET and PD patients reported a bidirectional interaction between the primary sensorimotor area of cortex and the affected muscles, with an efferent and afferent delay between 10 and 30 ms (7–10, 13). In our dataset, the EEG activity preceded the motor unit firings in half of the cases, and in the other half followed it. This is likely due to the fact that the primary motor and sensory cortices are next to each other and the limited spatial resolution of the EEG makes their activities hard to disentangle. We want to emphasize that these results were obtained using significantly shorter datasets (30 s vs. the typically ≥60 s long signals employed in other studies), and avoiding the need of manually discarding epochs with artifacts.

Results in Figure 6B suggest that the strength of our method derives from the direct use of the CST in the identification of the tremor-related cortical activity. One of the reasons for this is that the CST provide a more accurate representation of the common synaptic input to the muscles than rectified EMG as it eliminates the influence of frequency components introduced by the motor unit action potentials (22, 44). In the case of tremor, the CST has most of its power at the frequency of the tremor and its harmonics (15, 45). Thus, our approach averages out artifacts and other non-physiological factors (42).

Our corticospinal latency results are consistent with previous studies (7–10, 13). However, they must be interpreted with caution. The convolutive mixing models used to represent the EMG and EEG recordings, which are critical for accurate source separation (25, 27, 42), may introduce a temporal uncertainty to the reconstructed spike trains and tremor-related EEG components. We estimate this uncertainty to be about ±5 ms for each reconstructed source. Moreover, the propagation of the motor unit action potentials along the muscle fibers from the innervation zone to the uptake electrodes may introduce additional few ms delay. This could potentially further decrease the accuracy of EEG-CST delay estimation. In the current study, we used arrays of several tens of surface electrodes, whereas many previous studies were based on bipolar EMG recordings. The propagation of the motor unit action potentials may differ substantially across these two setups, and may also be muscle specific. Thus, the delays estimated in our study cannot easily be compared to the ones in other studies.

The availability of our method to automatically assess the accuracy with which each motor unit spike train is identified is also of critical importance because this accuracy is then reflected in the extracted tremor-related EEG component (see Appendix). Our group demonstrated in Holobar et al. (42) that motor units with PNR > 30 dB exhibit accuracy > 90% in identification of their firing patterns and in this study, we carefully utilized this knowledge to increase the accuracy of EEG component identification. In the future, we will investigate the minimal number of EEG channels required for accurate detection of tremor-related EEG activity, since it is likely that not all the EEG channels included in this study contribute significantly to tremor identification.

The proposed method is also computationally efficient. The most time consuming step is its first stage (surface EMG decomposition), which typically requires a few minutes of processing time on regular PC for 30 s long measurements. EEG decomposition in Equations (10, 11) is performed quickly.

The method does require multichannel EMG recordings from a muscle, increasing the experimental costs. However, multichannel EMG acquisition demonstrated significant progress in the recent years and became an important source of information in neurophysiology, neurology, sport sciences, prosthetics and ergonomics, to name just a few major scientific fields. Thus, it is likely that the price of multichannel acquisition systems will decrease in the near future.

We limited our study to the EEG decomposition of pathological tremor. The latter is a specific neurological disorder that is characterized by clear spectral peaks in acquired EEG, EMG, and inertial data. It is currently unclear to what extent the presented methodology is applicable to investigations of other types of pathological tremor (e.g., dystonic or cerebellar tremor) or to other disorders, such as multiple sclerosis, stroke and traumatic brain injuries and overactive thyroid, especially as tremor frequently accompanies these disorders. All these questions need to be systematically addressed in separate studies.

In conclusion, we have presented a novel method for estimating tremor-related cortical activity. This method uses pooled motor unit firings to directly extract the tremor component from cortical recordings. Based on the presented results, we believe that our method constitutes a significant step forward in the current state-of-the-art as: (a) it is the first method that directly extracts the tremor component from EEG recordings; (b) it successfully tracks time changes in the tremor-related cortical activity and has a potential for online tremor detection.

## Author contributions

AH, JG, ER, JR, JB-L, JP, and VG participated in conceptualization and data acquisition. AH, JK, and VG participated in formal analysis and methodology. AH, ER, JB-L and JP participated in project administration and resources. All authors contributed to writing the original draft and revised the manuscript. All authors read and approved the final version of the manuscript and agreed for all aspects of the work.

### Conflict of interest statement

The authors declare that the research was conducted in the absence of any commercial or financial relationships that could be construed as a potential conflict of interest.

## Appendix

### Estimation of ${\text{c}}_{{\text{s}}_{\text{j}}\left(\text{n}\right),\bar{\text{s}}}\;$ in the case of phase-locked motor unit firings

Assume that tremor-related EEG component in its analytic form can be expressed as a complex exponential function:

(A1) $$s_{j}\left(n\right)=e^{i\left(2\pi \frac{f_{j}}{f_{s}}n+\varphi_{j}\right)}$$

Where *i* is imaginary unit, *f*_*j*_ and ϕ_*j*_ are the frequency and the phase of *s*_*j*_, respectively, and *f*_s_ is the sampling frequency. Then the cross-correlation **c**_*s*_*j*_, *s*_λ__(*d*) can be approximated by the following sample mean:

(A2) $$\begin{array}{l} c_{s_{j},s_{\lambda }}(d)=\frac{1}{N}\sum_{n=0}^{N-1}s_{j}\left(n\right)s_{\lambda }^{H}\left(n+d\right)\\ \;=\frac{1}{N}\sum_{n=0}^{N-1}e^{i\left(2\pi \frac{f_{j}}{f_{s}}n+\varphi_{j}\right)}e^{-i\left(2\pi \frac{f_{\lambda }}{f_{s}}\left(n+d\right)+\varphi_{\lambda }\right)}\\ \;=\frac{1}{N}e^{i\left(\varphi_{j}-\varphi_{\lambda }-2\pi \frac{f_{\lambda }}{f_{s}}d\right)}\sum_{n=0}^{N-1}e^{i2\pi n\frac{f_{j}-f_{\lambda }}{f_{s}}} \end{array}$$

As well-known from Fourier analysis, the sum over *N* in (A2) converges to zero when *N* → ∞ and *f*_*j*_≠*f*_λ_. When *f*_*j*_ = *f*_λ_ (A2) yields

(A3) $$c_{s_{j},s_{j}}(d)=e^{-i2\pi \frac{f_{j}}{f_{s}}d}$$

Define now the *r*-th motor unit spike train as

(A4) $$t_{r}\left(n\right)=\sum_{k=0}^{K_{r}-1}\delta \left(n-k\frac{f_{s}}{f_{r}}-d_{r}-\Delta d_{rk}\right)$$

where *d*_*r*_ is the common time lag (phase) of the pulses in *t*_*r*_(*n*), Δ*d*_*rk*_ is the *k*-th realization of Gaussian random variable Δ*d*_*rk*_ ~ *N*(0, σ_Δ_*d*__*r*__), *f*_*r*_ is frequency of motor unit firings, and *K*_*r*_ is the number of firings in the observed time interval. Then

(A5) $$\begin{array}{l} c_{t_{r},s_{j}}\left(d\right)=\frac{1}{N}\sum_{k=0}^{K_{r}-1}e^{-i\left(2\pi \frac{f_{j}}{f_{s}}\left(k\frac{f_{s}}{f_{r}}+d_{r}+\Delta d_{rk}+d\right)+\varphi_{j}\right)}\\ \;=\frac{1}{N}e^{-i\left(2\pi \frac{f_{j}}{f_{s}}\left(d_{r}+d\right)+\varphi_{j}\right)}\sum_{k=0}^{K_{r}-1}e^{-i2\pi k\frac{f_{j}}{f_{r}}}\cdot e^{-i2\pi \frac{f_{j}}{f_{s}}\Delta d_{rk}} \end{array}$$

When EEG tremor is coupled to motor unit firings, we have *f*_*j*_ = *af*_*r*_ for *a ϵ* ℤ. Thus, $e^{-i2\pi k\frac{f_{j}}{f_{r}}}=1,\;\forall k,\;$ and (A4) simplifies to

(A6) $$c_{t_{r},s_{j}}\left(d\right)=\left(\frac{1}{N}e^{-i\left(2\pi \frac{f_{j}}{f_{s}}d_{r}+\varphi_{j}\right)}\right)\left(\sum_{k=0}^{K_{r}-1}e^{-i2\pi \frac{f_{j}}{f_{s}}\Delta d_{rk}}\right)e^{-i2\pi \frac{f_{j}}{f_{s}}d}$$

Since Δ*d*_*rk*_ ~ *N*(0, σ_Δ_*d*__*r*__) and σ_Δ_*d*__*r*__is relatively small with respect to *f*_*s*_, the imaginary component of the sum in (A6) converges to zero, so that $\overset{K_{r}-1}{\underset{k=0}{\sum }}e^{-i2\pi \frac{f_{j}}{f_{s}}\Delta d_{rk}}=\beta {\leq K}_{r}$ when *K*_*r*_ → ∞, where β is a real number. When EEG tremor is not coupled to motor unit firings, i.e., *f*_*j*_ ≠ *af*_*r*_ for *a ϵ* ℤ, (A5) converges to zero when *K*_*r*_ → ∞. Therefore, for sufficiently large *K*_*r*_ (A5) and (A6) yield:

(A7) $$c_{t_{r},s}\left(d\right)\approx \alpha c_{s_{j},s}\left(d\right)$$

with $\alpha =\frac{\beta }{N}e^{-i\left(2\pi \frac{f_{j}}{f_{s}}d_{r}+\varphi_{j}\right)}$. In practice, The higher the number of motor unit firings *K*_*r*_, the better the estimate (A6). We can increase *K*_*r*_ by increasing the length of the signal's time support, but this comes at the cost of long signal acquisitions. In the case of pathological tremor, motor unit firing patterns are highly synchronized and active motor units share approximately the same tremor-related firing rate *f*_*r*_ = *f*_*T*_, ∀*r* and initial delays *d*_*r*_ = *d*_*T*_, ∀*r* (see Figure 4). In such case, the CST of *R* motor units can be modeled by

(A8) $$CST(n)=\;\sum_{r=1}^{R}t_{r}\left(n\right)=\sum_{r=1}^{R}\sum_{k=0}^{K_{r}-1}\delta \left(n-k\frac{f_{s}}{f_{T}}-d_{T}-\Delta d_{rk}\right)$$

This increases the number of motor unit firings in (A5) to

(A9) $$\begin{array}{l} c_{CST,s_{j}}(d)=\left(\frac{1}{T}e^{-i\left(2\pi \frac{f_{j}}{f_{s}}d_{T}+\varphi_{j}\right)}\right)\left(\sum_{r=1}^{R}\sum_{k=0}^{K_{r}-1}e^{-i2\pi \frac{f_{j}}{f_{s}}\Delta d_{rk}}\right)\\ \;e^{-i2\pi \frac{f_{j}}{f_{s}}d} \end{array}$$

and, thus, improves the estimate (A6).

In noise-free conditions (A6), (A8) and (7) fully justify the approximation (9). In the presence of noise, however, further analytical derivations of the noise residual are required to verify whether the proposed estimator (10) is truly an LMMSE estimator and, thus, Bayesian optimal. Nevertheless, the results on both synthetic and experimental signals confirm the effectiveness of the proposed tremor estimation.
